# Postmortem skeletal muscle metabolism of farm animals approached with metabolomics

**DOI:** 10.5713/ab.22.0370

**Published:** 2022-11-14

**Authors:** Susumu Muroya

**Affiliations:** 1 Animal Products Research Group, NARO Institute of Livestock and Grassland Science (NILGS), Tsukuba, Ibaraki 305-0901, Japan

**Keywords:** Breed, Metabolism, Metabolomics, Muscle Fiber Type, Postmortem Aging

## Abstract

Skeletal muscle metabolism regulates homeostatic balance in animals. The metabolic impact persists even after farm animal skeletal muscle is converted to edible meat through postmortem rigor mortis and aging. Muscle metabolites resulting from animal growth and postmortem storage have a significant impact on meat quality, including flavor and color. Metabolomics studies of postmortem muscle aging have identified metabolisms that contain signatures inherent to muscle properties and the altered metabolites by physiological adaptation, with glycolysis as the pivotal metabolism in postmortem aging. Metabolomics has also played a role in mining relevant postmortem metabolisms and pathways, such as the citrate cycle and mitochondrial metabolism. This leads to a deeper understanding of the mechanisms underlying the generation of key compounds that are associated with meat quality. Genetic background, feeding strategy, and muscle type primarily determine skeletal muscle properties in live animals and affect post-mortem muscle metabolism. With comprehensive metabolite detection, metabolomics is also beneficial for exploring biomarker candidates that could be useful to monitor meat production and predict the quality traits. The present review focuses on advances in farm animal muscle metabolomics, especially postmortem muscle metabolism associated with genetic factors and muscle type.

## INTRODUCTION

Metabolites, a group of small biomolecules with hydrophilic and hydrophobic compounds mostly with molecular weights less than 1,000 Da, constitute the basic components of biological tissues. They are also important as phenotypic determinants of food materials in live animal and plant tissues because metabolites such as amino acids (AAs), lipids, and nucleotide phosphates function as flavor-related components after harvest [1]. Moreover, metabolites in biological tissues, including animal blood, represent phenotypic signatures of tissue samples, which can be utilized to monitor and evaluate tissue growth and product quality.

Skeletal muscle properties are determined by a functionally cooperative set of genes specific to the spatiotemporal requirements of each muscle [2]. In the process of muscle development, growth, and maturation, genes are expressed through regulation at the transcriptional, post-transcriptional, and translational levels, resulting in metabolite generation by multistep enzymatic reactions [3,4]. Even after tissue growth and maturation, the content of muscle metabolites is altered through gene expression and regulation based on physiological requirements, as observed in muscle type specification [5]. Skeletal muscle metabolite composition is a major factor in characterizing muscle properties, and thereby the nutritional and sensory properties of the meat; thus, metabolite composition provides important phenotypic information [6].

In the past decades, metabolomics has been applied in animal breeding, husbandry, processing, and postmortem storage research, owing to the high-throughput advantage of metabolite data acquisition [7–11]. Metabolomic profiling is very beneficial for comprehensively investigating the metabolite composition of a muscle sample and for understanding the impact of various endogenous and exogenous factors on live and postmortem muscle metabolism and subsequent meat quality [6,12].

In this context, the present review provides an overview of recent findings and topics of muscle and meat metabolism approached with metabolomics, which we call MEATabolomics [6], focusing on postmortem muscle metabolism. This review also outlines the recent metabolomic topics in postmortem muscle metabolism during aging. The impact of genetic background and muscle type on postmortem muscle metabolism is discussed in association with the meat quality traits of cattle, pigs, and sheep. Topics such as meat quality, spoilage, processing, authentication, and methodology can be found in a previous review [6].

## ANALYTICAL METHODS OF METABOLOMICS

Metabolites can be categorized into groups based on their physicochemical properties, such as hydrophobicity, hydrophilicity, and volatility. Currently, there are two major types of metabolomics techniques: nuclear magnetic resonance (NMR) [11] and mass spectrometry (MS) [13]. NMR is also highlighted as a practical method for analytical routines. This technique provides rapid and reproducible measurements of complex mixtures without time-consuming pretreatments. Although NMR has relatively low resolution and sensitivity compared to that of MS-based techniques, it can collect distinct information that other metabolomics cannot access in a non-destructive and non-biased manner [11].

In MS-based metabolomics, separation columns are selectively equipped with an MS measurement apparatus, depending on the hydrophobicity and polarity of the target compounds. In capillary electrophoresis (CE)–MS, polar and charged metabolites are effectively separated [14,15]. Compared with that of gas chromatography (GC) and liquid chromatography (LC), CE separation exhibits a higher resolution for ionic compounds (including their isomers) because of the separation of molecules by their charge-to-mass ratios. GC–MS has been employed for decades owing to its high GC separation efficiency and broad target metabolite range, including fatty acids (FAs) and sugars, even with the requirement of derivatization for non-volatile target compounds. In LC-MS, including high-performance LC (HPLC)-MS, compounds with lower polarity are targeted, but the target molecules can be flexibly changed by replacing the separation columns. In most cases, after separation in the column, molecules are ionized by electrospray ionization (ESI) in CE, electron ionization (EI) or chemical ionization (CI) in GC, and ESI or atmospheric pressure chemical ionization (APCI; API) in LC. Currently, there are a variety of MS detection techniques, of which the one most frequently used for matching the upstream CE, GC, and LC separation steps is time-of-flight (TOF). MS-based approaches are used for both targeted and untargeted metabolomics [16–18]. In recent years, rapid evaporative ionization mass spectrometry (REIMS) has been applied in meat metabolomics [16,19]. Based on ambient ionization, this method directly measures the MS of a biological sample without requiring preparative steps [20,21].

The steps of metabolomic data acquisition are followed by downstream statistical analysis and data mining, which includes various multivariate analyses, such as principal component analysis (PCA), hierarchical clustering analysis (HCA), partial least square analysis (also called “projection to latent structures” - PLS), PLS–discriminant analysis (PLS–DA), and random forests (RF) [22,23]. The differences between the classification methods are mentioned previously [6,24]. Data analyses can classify the samples statistically based on sample characteristics and determine metabolites responsible for the meaningful phenotype. This helps us identify biomarker candidates to monitor and assess food quality, as well as to elucidate molecular pathways of key metabolite generation by enzymes [22].

## POSTMORTEM MUSCLE AGING APPROACHED WITH METABOLOMICS

Postmortem muscle aging and storage significantly impact meat quality traits [25–27]. Development of flavor and tenderness in meat due to postmortem aging has a major impact on consumers’ purchasing decisions [28]. Even when an animal has a high-quality potential for muscle as meat, the final meat quality might be lowered by inappropriate management, resulting in deterioration such as spoilage and discoloration accompanying abnormal metabolome changes. Inversely, postmortem meat aging under the appropriate conditions greatly benefits the final meat quality, such as flavor development [1] without unfavorable metabolite alteration. Moreover, various factors such as species, breed, animal age, diet, individual muscle, and marbling content could have distinct influences on the rate and extent of the postmortem aging response of skeletal muscle and subsequent meat quality, including tenderness and flavor [26,29,30]. Postmortem muscle aging is also affected by environmental conditions, such as temperature and length of the period, accompanied by alterations in metabolisms [27,31,32].

During the postmortem aging of meat, the energy substrate or oxygen is no longer supplied to cells from the outside. ATP is drastically consumed and finally exhausted within 24 h post-mortem in bovine [32] and porcine muscles [33] for cell survival. In such an anaerobic environment, muscle cells manage to survive, depending on glycolysis, owing to the inability of oxidative phosphorylation. Glycogen and other glucose-related metabolites are mobilized into glycolysis as energy substrates [34], resulting in lactate accumulation and subsequent pH decline [35]. Consequently, many cytosolic and myofibrillar proteins, such as enolase, troponin T, and desmin, are exposed to acidic denaturing environments. Neutral proteases, calpains, are activated by Ca^2+^ released from the dysregulated sarcoplasmic reticulum, while caspases also participate in the postmortem degradation of muscle cell proteins [25,27]. Muscle protein degradation is thought to contributes to postmortem meat tenderization [27] and the generation of flavor component precursors, such as AAs and peptides [26]. In early postmortem, ATP is also rapidly consumed, which causes temporal accumulation of inosine 5′-monophosphate (IMP), one of the major components of *umami* flavor [36–38]. These biochemical changes lead to tenderness and flavor development during postmortem aging, consequently affecting palatability and consumer satisfaction [28]. Some of these compounds, such as AAs, FAs, lipids, and carbonyl compounds, are precursors of aroma compounds developed through the Maillard reaction and oxidation, particularly during the heating process [26,39,40].

Moreover, glycolysis causes denaturation of hydrophilic proteins [35] through a decline in pH [41]. This partly leads to a reduction in water holding capacity (WHC) and thereby loss of moisture in meat [42,43], although the relationship between WHC and sensory juiciness remains unclear [25]. Nevertheless, WHC is improved in long-term aged meat, suggesting the effect of proteolytic activity on postmortem intracellular spaces for water molecules to be retained in the cell [44–46]. This suggests a considerably complicated interplay of glycolysis and protein degradation changes in WHC. Thus, biochemical changes in postmortem muscle, especially protein and energy substrate metabolism, have been intensively investigated to improve meat quality, such as tenderness and juiciness. However, changes in diverse metabolites and their networks have been poorly elucidated, even with the potential contribution of these compounds to meat quality traits.

**Figure 1:**
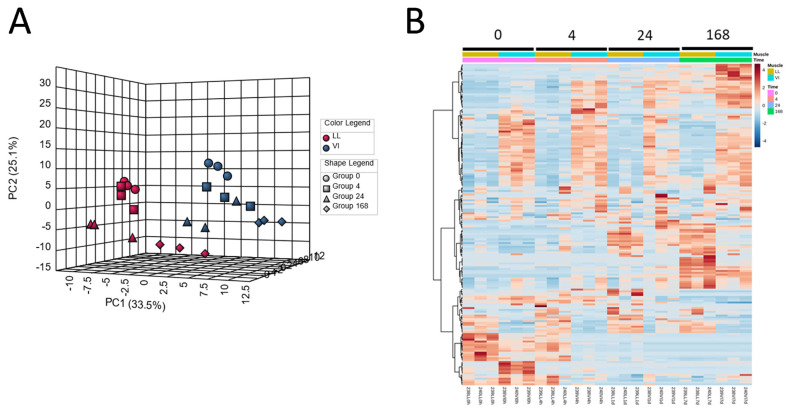
Classification of pig LL and VI muscle samples at different postmortem aging periods. Pig LL and VI muscles were aged for 0, 4, 24, 168 h postmortem. (A) Result of PCA, (B) heatmap result of HCA. LL, *longissimus lumborum*; VI, *vastus intermedius*; PCA, principal component analysis; HCA, hierarchical clustering analysis.

Metabolomics has been applied to postmortem meat aging studies to account for the impacts of postmortem aging, mainly on meat quality in terms of metabolites. In pork, postmortem changes in the *longissimus lumborum* (LL) and *vastus intermedius* (VI) muscles were investigated using a CE–MS approach [33]. In this study, the levels of numerous muscle metabolites were altered, suggesting that postmortem muscle metabolism was drastically altered, especially within 24 h of slaughter. The PCA and HCA results explicitly show marked differences in the metabolite distribution of samples between postmortem aging times and muscle types (Figure 1). Intermediate glycolytic products, glucose 6-phosphate (G-6P) and fructose 6-phosphate (F-6P), increased in the LL samples until 24 h postmortem, while downstream compounds such as fructose 1,6-bisphosphate (F-1,6P) and phosphoenolpyruvate decreased with exhaustion at 24 h postmortem, indicating the rate-determining activity of phosphofructokinase (F-6P → F-1,6P) in postmortem LL muscle glycolysis. Additionally, in the LL muscle, most of the AAs and identified dipeptides increased after day 1 of aging, suggesting that protein degradation began to be dominant after day 1. In ATP degradation, changes in the product levels were observed along the ADP → AMP → IMP → inosine → hypoxanthine pathway, with a time lag between the products. Similar results were also observed in early postmortem [47], during 14 days of aging [48,49], and in long-term aging of beef [50,51]. In these studies, metabolism associated with purine, pyrimidine, glutathione, nicotinate, glycerophospholipid, pentose phosphate, and AAs was found to be a prominent biological pathway in postmortem muscle aging [48,49]. Moreover, other metabolisms related to nutrients, such as choline, nicotinamide, and thiamine, also begin to occur in pork [33] and beef [48, 49]. Most of these pathways were commonly observed in pig and bovine muscles in these studies, indicating that these metabolic pathways are essential for postmortem muscle metabolism. In addition, several citrate cycle metabolites and enzyme cofactors decreased within 24 h postmortem, accompanied by alterations in glycolytic and purine metabolite levels [47]. This may be due to mitochondrial damage during post-mortem muscle aging. Studies focusing on mitochondria have suggested the involvement of reactive oxygen species (ROS) from dysregulated mitochondria in early postmortem muscle aging [52–54]. According to these results, ROS-induced postmortem mitochondrial degeneration leads to increased drip loss in beef meat. Such mitochondrial damage in early postmortem may induce muscle cell apoptosis, which potentially affects the conversion of muscle to meat [55,56], and thereby the meat quality, such as meat discoloration by the oxidation of FAs and lipids [57].

**Figure 2:**
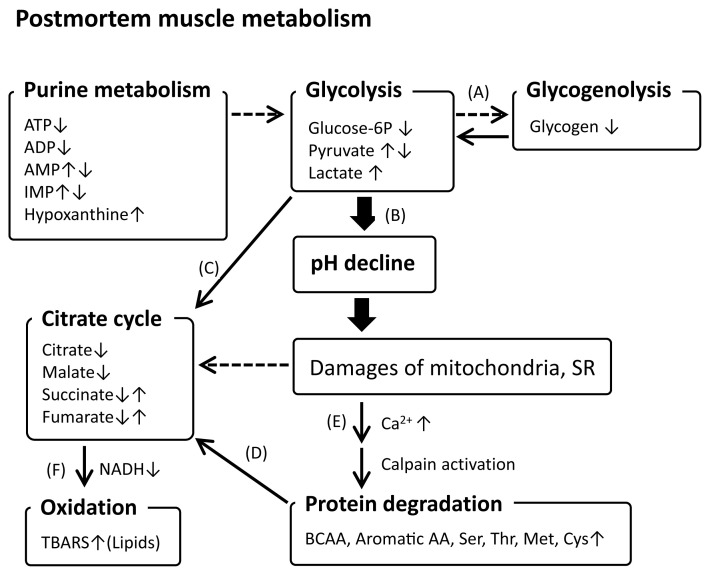
A hypothetical representation of postmortem muscle metabolisms progressing during the aging of beef and pork. BCAA, branched chain amino acid; TBARS, 2-thiobarbituric acid reactive substances. Broken chain arrows indicate potential induction of the directed metabolism by a progressing metabolism. ↑ and ↓ indicate increase and decrease, respectively. References: (A) [42,57,58], (B) [32,46], (C) [46,47,59], (D) [60], (E) [61,62], (F) [56,59].

Thus, postmortem muscle metabolism progresses in an organized manner (Figure 2). This process is initiated by energy substrate shortage and subsequent glycolysis with the stimulation of glycogenolysis [43,58,59], leading to lactate accumulation and pH decline [35]. Postmortem muscle glycolysis largely affects the downstream biochemical changes associated with meat quality [31], which have been recently unveiled by metabolomics studies. Metabolomics has exerted its power to mine the relevant metabolisms and pathways that are minor in postmortem muscle aging and thereby have never been unveiled so far, other than glycolysis and purine metabolism. For example, pyruvate temporally accumulated by glycolysis is likely mobilized to the citrate cycle [47,48,60] to generate nicotinamide adenine dinucleotide (NADH), a major reducing metabolite. The changes in metabolites in the citrate cycle, fumarate, citrate, succinate, and malate, during postmortem aging [47,48] also suggest the contribution of the citrate cycle to NADH regeneration. The resultant reduced cellular conditions are critical for protecting intracellular molecules against an oxidizing environment. In terms of NADH generation, AAs generated by protein degradation are important sources of citrate cycle materials [61], which may be induced by decrease in energy substrates. Acidification of the intracellular environment by pH decline is thought to result in calpain activation by increased Ca^2+^ concentration from the dysregulated sarcoplasmic reticulum and caspase-3 activation, possibly via mitochondrial apoptosis-related pathways [62,63], and may release cathepsins from lysosomes. Activated proteases degrade proteins that contribute to AA and peptide production. ATP degradation to ADP and related metabolites, originating from energy demand for cell survival, could be linked with energy generation-related metabolism, including glycolysis and the citrate cycle [43,47,48]. This could also be true for postmortem autooxidation, which occurs on lipids and proteins in association with mitochondrial degeneration and NADH depletion [57,60].

Changes in meat metabolite set depend on postmortem handling of meat. Dry aging allows moisture evaporation under aerobic conditions with a relatively long aging period to obtain a beneficial dry-aged flavor [25]. A dry aged beef under a condition of 3°C with 0.2 m/s air velocity for 3 weeks improved the beef palatability in a sensory panel evaluation, compared with a conventional wet aging and other dry storage conditions, with higher branched chain amino acids (BCAAs) and IMP contents [64]. The airflow in this method might simply increase flavor-associated metabolites by water evaporation; however, other unknown factors are also likely to be involved. As a major factor affecting metabolites during dry aging of pork loins, microorganisms seem to participate in generation of aroma-associated compounds [65]. Effect of microorganisms on flavor compound generation in dry aging was also observed in pork loins, which was partly cancelled (regarding phenylalanine and benzyl benzoate) when the meat was exposed to ultraviolet light [66]. These results suggest that microorganisms can be utilized for development of meat aroma and flavor. On the other hand, avoiding of negative effect of microorganisms has been attempted by using a water-permeable bag (in-bag dry aging technique), in which the effect of the in-bag dry aging on lamb muscles was assessed using a metabolomic approach [67]. During 21 days of aging, metabolomic differences were observed between the two aging methods and muscles [67]. Dry-aged lambs showed higher levels of AAs, dipeptides, and energy metabolism-related metabolites than those in wet-aged lambs. According to the OPLS-DA model using REIMS data, differences between the two different types of aging methods and different types of muscles were distinguished. Glutathione metabolism was extracted as a specific pathway for conventional dry-aged beef in the pathway analysis. Like traditional dry aging methods, this method allows moisture in the meat to evaporate during aging but prevents microbial contamination (and consequent loss by excessive trimming) without compromising the flavor of the aged meat [68]. Thus, dry aging may alter postmortem metabolism in beef and lamb by reducing or oxidizing muscle in response to an oxidizing environment.

The metabolites showing significant changes or differences between meat storage conditions or between muscles with different backgrounds of animal production can be screened by multivariate analyses, including PCA and PLS-DA. With the confirmed association of a candidate metabolite with a specific trait or phenotype, biomarker candidates for meat quality traits can be identified from biologically meaningful metabolites compared with target traits, such as the rate and extent of oxidation [57] or aging [48]. Although the candidate needs to be validated for practical use, the candidate biomarker metabolite could potentially be useful for monitoring and controlling the quality of meat production processes.

## MUSCLE TYPE AND GENETIC FACTORS AFFECTING POSTMORTEM MUSCLE MEABOLISMS

The skeletal muscle type has a significant impact on postmortem aging. Skeletal muscle tissue is fabricated with a hierarchical myofiber bundle structure. Myofibers are classified into two major types, fast and slow, based on their contractile and metabolic properties with specific markers, such as myosin heavy chain (MYH) isoforms [69]. This reflects the differential expression of genes and proteins between different types of myofiber cells [69–73]. Accordingly, fast- and slow-type muscles with varying compositions of fast- and slow-type myofibers exert distinct physiological functions. Previous studies based on gene and protein expression have suggested that metabolite composition is considerably different between physiologically different types of muscles or myofibers, which also suggests intermuscular differences in postmortem metabolism during aging. Indeed, differences in metabolite distribution were observed between different muscle types: an abundance of AAs (Ala, Gly, Gln, and His) and glutathione in porcine VI muscle (a slow-type muscle) and abundance of γ-aminobutylate, nucleotide sugars, and anserine in LL muscle (a fast-type muscle) immediately after slaughter [33]. The essential postmortem metabolism, such as glycolysis, purine metabolism, and AA degradation, differ in the rate and extent in relevant pathways between fast- and slow-type muscles.

Thiamine phosphate metabolism also differ between VI and LL muscles during postmortem aging of pork [73]. Thiamine triphosphate (ThTP), an abundant thiamine derivative specifically in porcine muscle, is converted to thiamine through thiamine diphosphate (ThDP) accumulation 24 h postmortem, with a difference between the two muscle types. In addition to the coenzymatic role of ThDP, thiamine and its derivatives are also known to participate in biological responses to oxidative stress, both as ROS scavengers [74] and regulators of gene expression. Furthermore, ThTP and adenylate ThTP activate bovine liver glutamate dehydrogenase, which generates 2-oxaloacetate from glutamate to provide citrate cycle substrates [75]. However, the roles of thiamine and its derivatives in postmortem muscle metabolism require further investigation. Intermuscular differences in postmortem metabolite distributions have also been observed in bovine studies focusing on early postmortem energy metabolism [47] and lipid oxidation [57]. In addition, the content of glycerophospholipid, an essential compound of the mitochondrial membrane, differed between the *longissimus dorsi* (LD) and *psoas major* (PM) muscles in early postmortem [54]. This could be explained by varying contents of mitochondria between the two muscle types [76] accelerated the oxidation of glycerophospholipids, and thereby, damaged the mitochondria. Since mitochondria play a crucial role in energy generation through β-oxidation and the citrate cycle, in connection with glycolysis and AA metabolism, all metabolisms related to energy substrates and sources likely differ between slow and fast muscle types.

Genetic factors are crucial determinants of meat quality traits [77], which are potentially affected by postmortem muscle aging. To date, differences in muscle metabolome profiles between breeds or genotypes have been observed in bovine, porcine, and sheep muscles [6]; however, most of these were detected in muscle samples at several days postmortem without comparison between before and after muscle aging. Nevertheless, a pork study investigating genetic differences associated with the extent of drip loss was conducted using an integrative metabolomics approach with genomics [18]. This study employed a single nucleotide polymorphism-based genome-wide association study (GWAS). In the results, an enrichment analysis resulted in 10 pathways, including sphingolipid metabolism and glycolysis/gluconeogenesis, with significant influence on drip loss and identification of a region of candidate genes on chromosome 18 as being associated with drip loss and the metabolite glycine. In integrative omics studies, such as GWAS, metabolomics greatly contributes to the capture of phenotypic metabolites and candidate responsible genes.

**Figure 3:**
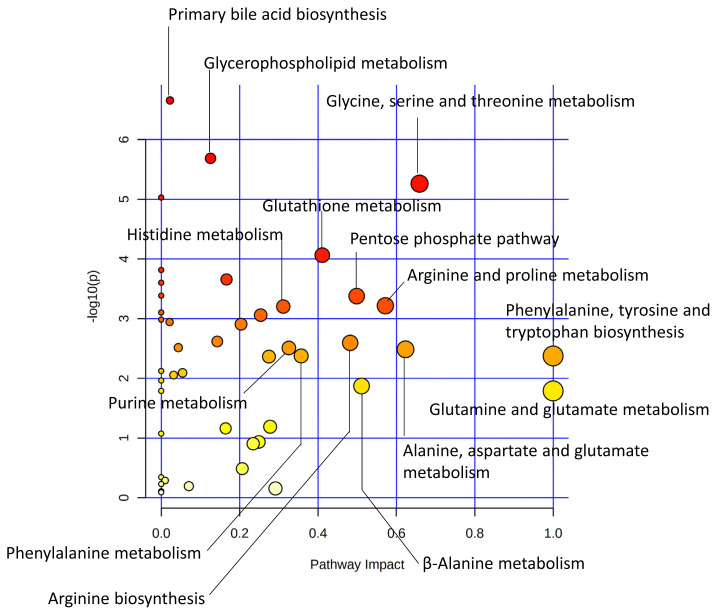
Different metabolic pathways between JBRT and JB *longissimus* muscle at day 14 postmortem. The metabolic pathways are analyzed based on absolutely-quantified content of 104 metabolites [49]. This metabolome view shows highly scored pathways according to the p values from the pathway enrichment analysis (vertical axis) and pathway impact values from the pathway topology analysis (horizontal axis). A pathway with small p value and high impact is plotted as a red and large circle. JB, Japanese Black; JBRT, Japanese Brown (the Kochi pedigree).

In a study comparing Japanese Black (JB) and Japanese Brown (JBRT, the Kochi pedigree) cattle, lower lactate content and higher ultimate pH were observed in JBRT than in JB in aged beef [49], which was one of the prominent interbreed differences in the postmortem metabolisms (Figure 3). This indicates a lower glycolytic rate and activity in JBRT. Accumulation of F-1,6P at 14 days postmortem was higher in JBRT than in JB beef, without differences in G-6P and F-6P levels, indicating that the slower glycolysis in JBRT is due to the lower consumption of F-1,6P in JBRT than in JB [49]. Moreover, in JBRT beef, IMP accumulation from ATP degradation was higher but AA accumulation was lower than in JB beef, which suggests that lower postmortem glycolysis and subsequent pH decline might affect these metabolisms in JBRT beef. However, most of the cytosolic enzymes responsible for these metabolic processes are presumed to be susceptible to low pH, possibly resulting in reduced activity in postmortem muscle cells. Although these differences in postmortem metabolism between the two breeds require further investigation, significantly different metabolites, such as F-1,6P, are considered as candidate biomarkers to distinguish the two cattle breeds.

Dietary composition and feeding conditions, including the environment, are crucial modulatory factors in skeletal muscle metabolism. Indeed, a number of studies have been intensively conducted in cattle, pigs, chickens, and sheep to investigate the effect of dietary supplements (*e.g.*, lysine and antioxidants such as mate extract [78,79]) or feeding management (*e.g.*, grazing [80]) on meat quality traits and the relevant metabolites [6]. Nevertheless, little is known about live muscle metabolism and much less is known about postmortem muscle metabolism. Details of the effects of feeding and diet on postmortem metabolism in the muscles require intensive investigation.

## CONCLUSIONS AND PERSPECTIVES

Skeletal muscle metabolism is basically determined by a genetic program through the developmental process, but afterward, it is specified depending on muscle type, being modulated by the environment with physiological adaptation. Muscle metabolites drastically change during postmortem aging, which is inherent in live muscle metabolism. Metabolomics studies in the last decade have provided comprehensive information on metabolite networks, which has expanded and deepened the understanding of postmortem metabolism with glycolysis, AA generation, and purine metabolism. Postmortem muscle aging depends primarily on these major metabolic pathways. Recent metabolomics studies have further revealed that the citrate cycle and redox-related metabolism, including those of glutathione and NADH, also change during postmortem aging and could be linked with glycolysis and protein degradation. Such metabolic changes significantly impact the flavor, WHC, and color of meat. Postmortem muscle metabolism is affected by muscle type and genetic factors, including animal breed. The feeding environment and diet also have the potential to alter live muscle metabolism, through which postmortem metabolite levels are changed; however, the mechanism underlying this effect needs to be further investigated. When comparing different animal breeds, muscle types, feeding, or postmortem aging methods, metabolites as biomarker candidates can be determined by metabolomics to distinguish differences in those conditions.
